# The Significance of Serum C-Reactive Protein and Neutrophil–Lymphocyte Ratio in Predicting the Diagnostic Outcomes of Renal Mass Biopsy Procedure

**DOI:** 10.15586/jkcvhl.v10i1.259

**Published:** 2023-02-03

**Authors:** Aykut Colakerol, Sergen Sahin, Ramazan Omer Yazar, Mustafa Zafer Temiz, Emrah Yuruk, Engin Kandirali, Atilla Semercioz, Ahmet Yaser Muslumanoglu

**Affiliations:** Department of Urology, Bagcilar Training and Research Hospital, Istanbul, Turkey

**Keywords:** cancer, C-reactive protein, biopsy, kidney, lymphocytes, neutrophils

## Abstract

This study aimed to investigate the predictive role of serum C-reactive protein (CRP) and neutrophil-to-lymphocyte ratio (NLR) on renal mass biopsy outcomes. A total of 71 patients with suspected kidney masses who underwent renal mass biopsy procedure between January 2017 and January 2021 were retrospectively evaluated. Pathological results after the procedure were obtained and pre-procedural serum CRP and NLR levels were extracted from the patients’ data. The patients were grouped into benign and malignant pathology groups according to the histopathology results. The parameters were compared between the groups. Diagnostic role of the parameters in terms of sensitivity, specificity, and positive and negative predictive values was also determined. Additionally, Pearson correlation analysis, and univariate and multivariate cox proportional hazard regression analyses were also performed to investigate the above association with tumor diameter and pathology results, respectively. At the end of the analyses, a total of 60 patients had malignant pathology on histopathological investigations of the mass biopsy specimens, whereas the remaining 11 patients had a benign pathological diagnosis. Significantly higher CRP and NLR levels were detected in the malignant pathology group. The parameters positively correlated with the malignant mass diameter, as well. Serum CRP and NLR determined the malignant masses before the biopsy with sensitivity and specificity of 76.6 and 81.8%, and 88.3 and 45.4%, respectively. Moreover, univariate and multivariate analyses showed that serum CRP level had a significant predictive value for malignant pathology (HR: 0.998, 95% CI: 0.940–0.967, P < 0.001 and HR: 0.951, 95% CI: 0.936–0.966, P < 0.001, respectively). In conclusion, serum CRP and NLR levels were significantly different in patients with malignant pathology after renal mass biopsy compared to the patients with benign pathology. Serum CRP level, in particular, diagnosed malignant pathologies with acceptable sensitivity and specificity values. Additionally, it had a substantial predictive role in determining the malign masses prior the biopsy. Therefore, pre-biopsy serum CRP and NLR levels may be used to predict the diagnostic outcomes of renal mass biopsy in clinical practice. Further studies with larger cohorts can prove our findings in the future.

## Introduction

The current standard management of kidney cancer is surgical resection of the renal malignant mass with nephrectomy. Patients underwent radical or partial nephrectomy procedures after the diagnosis, commonly made incidentally, with radiological imaging procedures (1, 2). However, the accurate method of kidney cancer diagnosis is still the histopathological analysis of the masses that can only be performed after surgical resection (3). On this point, pretreatment diagnostic histopathological investigation with renal mass biopsy comes into mind. Historically, renal mass biopsy has been used for a limited number of indications such as to diagnose unresectable kidney cancer and diagnose kidney cancer in patients who are poor surgical candidates (1). Major reason for those limited indications is the concern about the efficacy and safety of the renal mass biopsy (4). However, with recent technical advancements in the procedure, the use of renal mass biopsy procedure has started to increase in routine clinical practice (5). Several recent reports have described the safety and efficacy of the procedure (1). It seems that, in the near future, renal mass biopsy procedure may be used as a standard diagnostic method in selected cases. In this regard, predictive parameters for malignant renal masses prior to renal mass biopsy are needed today. However, to our knowledge, few studies have investigated the role of any predictive parameter for the outcomes of renal mass biopsy.

In this study, we investigated the predictive role of serum inflammatory markers, C-reactive protein (CRP), and neutrophil-to-lymphocyte ratio (NLR), in predicting the diagnostic outcomes of renal mass biopsy.

## Materials and Methods

A total of 71 patients with suspected kidney mass who underwent renal mass biopsy procedures between January 2017 and January 2021 were retrospectively evaluated. The approval for the study protocol was obtained from the Institutional Review Board (Approval ID: 2022/11/14/035). Informed patient consent was also obtained, and the study protocol was conducted according to the World Medical Association Declaration of Helsinki. Patients who were suspected of kidney mass without a previous kidney cancer history were included in the study. Patients with a history of any other malignancies, any anemia, active inflammatory and/or rheumatic diseases, and any acute infections were excluded.

An experienced interventional radiologist performed all biopsy protocols with an 18-gauge tru-cut biopsy needle under the guidance of ultrasonography (Logiq™ P6 Ultrasound Device, General Electric Co., NY, USA) imaging with a 3.5 MHz transducer after a local anesthesia with 1% lidocaine application. The separate tru-cut sampling technique was used and up to four cores were obtained from each kidney mass using an automatic 18-gauge biopsy needle (Estacore Automatic Biopsy Needle, GEOTEK Healthcare Products Co., Ankara, TR). Post-biopsy images were not routinely obtained, and patients were observed closely and were hemodynamically monitored for 3 h.

Characteristics of kidney masses including size, side, polarity, localization, and exophytic or endophytic nature were assessed by cross-sectional imaging studies. Pathological results after the procedure were obtained from the enrolled data, and pre-procedural serum CRP and NLR levels were extracted from patients’ data. The patients were grouped as benign and malignant pathology groups according to the histopathology results after the renal mass biopsy. Patient characteristics and the parameters were compared between the groups. The association of serum CRP and NLR levels with mass diameter in the malignant pathology group was also investigated. Univariate and multivariate cox proportional hazard regression analyses were also performed to investigate the above association of with malignant pathology results after the renal mass biopsy procedure.

Statistical analysis was performed with SPSS Version 22.0 Statistic Software Package (IBM SPSS Inc., Chicago, IL). Data distributions and test of normality were evaluated with the Shapiro–Wilk test. Descriptive statistic methods (mean ± standard deviation [SD] and percentages) were used to express data. We compared the normally distributed data between the groups using the independent t-test. The chi-square test was also used in the comparison of the nonparametric categorical variables. The Pearson correlation analysis was used to describe the association of the parameters with malignant mass diameter. Diagnostic ability and positive and negative predictive values of the parameters were determined with two-by-two sensitivity and specificity tables, at their most accepted cutoff levels by the literature. Univariate and multivariate cox proportional hazard regression analyses were also performed for each parameter to estimate their predictive role in determining the malignant pathology after the renal mass biopsy procedure. Differences were considered as significant at two-sided P < 0.05 and 95% confidence interval.

## Results

The mean age and mass diameter were 63.86 ± 12.93 years and 57.29 ± 36.23 mm, respectively. Out of the 71 cases, 43 (60.56%) were males and 28 (39.44%) were females. Comorbid diseases (any type) were detected in 38 (53.52%) cases, and 18 of them had multiple comorbidities. Out of the 71 cases, 11 (15.50%) had benign pathological findings after the renal mass biopsy. Five (45.5%) of them were reported as benign tissue without malignancy. Oncocytoma and angiomyolipoma were reported in four (36.4%) and two (18.1%) cases, respectively. Malignant pathological findings were reported in 60 cases (84.50%). Detailed information about the patients and the renal mass characteristics of patients with benign and malignant pathologies after the renal mass biopsy procedure are provided in Table 1.

**Table 1:** Patients and mass characteristics of the groups.

	Benign pathology group (n = 11)	Malignant pathology group (n = 60)	P
**Age (mean ± SD) (years)**	62.45 ± 12.13	64.10 ± 13.22	0.68*
**Gender** Male (n) Female (n)	5 6	38 22	0.21**
**Mass diameter (mean ± SD) (mm)**	36.36 ± 20.46	57.79 ± 35.45	0.01*
**Mass localization** Left (n) Right (n)	7 4	24 36	0.11**
**Mass localization** Anterior (n) Posterior (n) Medial (n)	4 5 2	23 25 12	0.74**
**Mass localization** Superior pole (n) Middle pole (n) Inferior pole (n) Whole kidney (n)	4 3 4 0	17 18 17 8	0.60**
**Exophytic mass nature** No (n) Yes (n)	8 3	41 19	0.02**
**Comorbidity** DM (n) HT (n) KAH (n) CRF (n)	2 4 3 2	13 26 15 6	0.32**

SD, standard deviation. *Independent t-test; **Chi-squared test.

**Figure 1:**
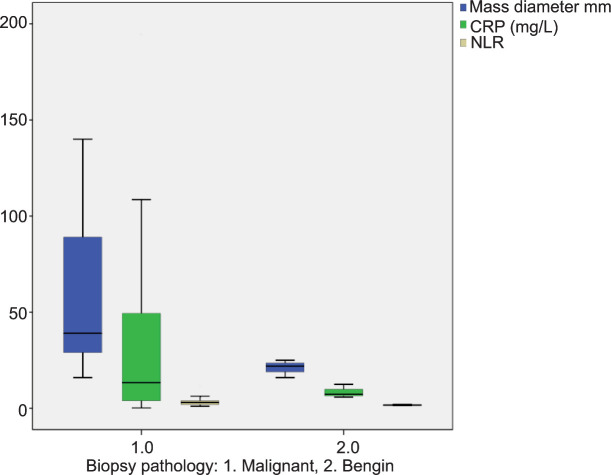
Levels of the parameters and tumor diameter between the groups within the graph.

The mean ages were 62.45 ± 12.13 versus 64.10 ± 13.22 years for patients with benign and malignant pathology after the renal mass biopsy, respectively (P = 0.68). The mean mass diameters were 36.36 ± 20.46 versus 57.79 ± 35.45 mm for the above patients, respectively (P = 0.01). The groups were similar in terms of the comorbid diseases and mass characteristics (Table 1). Serum CRP and NLR levels were significantly different between the groups. They were significantly higher (41.14 ± 51.82 vs 9.84 ± 3.69 mg/L, P = 0.002 and 3.46 ± 2.36 vs 1.54 ± 0.16, P < 0.001, respectively) in patients with malignant pathology compared to patients with benign pathology after the biopsy (Table 2 and Figure 1). Additionally, they were associated with the malignant mass diameter with correlation coefficients of 6.17 and 5.01, respectively (P < 0.001 and P = 0.001, respectively). With the cutoff value of 10 mg/L, serum CRP predicted the malignant masses before biopsy with a sensitivity and specificity of 76.6 and 81.8%, respectively (Table 3). On the other hand, serum NLR levels predicted the malignant masses with a sensitivity and specificity of 88.3 and 45.4%, respectively, at cutoff value 2.1 (Table 4). Moreover, univariate and multivariate analyses showed that serum CRP levels had a significant predictive value for malignant pathology (HR: 0.998, 95% CI: 0.940–0.967, P < 0.001 and HR: 0.951, 95% CI: 0.936–0.966, P < 0.001, respectively; Tables 5 and 6).

**Table 2:** Comparison of serum C-reactive protein and neutrophil-to-lymphocyte ratio levels between the groups.

	Benign pathology group (n = 11)	Malignant pathology group (n = 60)	P
**CRP, mg/L (mean ± SD)**	9.84 ± 3.69	41.14 ± 51.82	0.002*
**NLR (mean ± SD)**	1.54 ± 0.16	3.46 ± 2.36	<0.001*

CRP, C-reactive protein; NLR, neutrophil-to-lymphocyte ratio; SD, standard deviation. *Independent t-test.

**Table 3:** Sensitivity and specificity of C-reactive protein in predicting the malign pathology after renal mass biopsy.

	Benign pathology (n)	Malignant pathology (n)
**CRP ≤ 10 mg/L (n)**	9	14
**CRP > 10 mg/L (n)**	2	46

Sensitivity: 46/60 = 76.6%; Specificity: 9/11 = 81.8%; Positive predictive value: 46/48 = 0.95; Negative predictive value: 9/23 = 0.39. CRP, C-reactive protein.

**Table 4:** Sensitivity and specificity of neutrophil-to-lymphocyte ratio in predicting the malign pathology after renal mass biopsy.

	Benign pathology (n)	Malign pathology (n)
**NLR ≤ 2.1 (n)**	5	7
**NLR > 2.1 (n)**	6	53

Sensitivity: 53/60 = 88.3%; Specificity: 5/11 = 45.4%; Positive predictive value: 53/59 = 0.89; negative predictive value: 5/12 = 0.41. NLR, neutrophil-to-lymphocyte ratio.

**Table 5:** Univariate Cox Proportional Hazard Regression Analysis of the parameters age, gender, mass diameter, and serum CRP and NLR levels for prediction of malignant pathology after renal mass biopsy.

	HR	95% CI	P
**Age, years**	0.993	0.974–1.012	0.45
**Gender**	1.414	0.811–2.463	0.22
**Mass diameter, mm**	0.998	0.990–1.006	0.69
**Serum CRP, mg/L**	0.954	0.940–0.967	<0.001*
**Serum NLR**	0.881	0.747–1.039	0.13

* P < 0.05; CI: confidence interval; CRP, C-reactive protein; HR, hazard ratio; NLR, neutrophil-to-lymphocyte ratio.

**Table 6:** Multivariate Cox Proportional Hazard Regression Analysis of the parameters age, gender, mass diameter and, serum CRP and NLR levels for prediction of the malign pathology after renal mass biopsy.

	HR	95% CI	P
**Age, years**	0.999	0.997–1.022	0.94
**Gender**	1.391	0.736–2.627	0.30
**Mass diameter, mm**	1.003	0.994–1.012	0.50
**Serum CRP, mg/L**	0.951	0.936-0.966	<0.001*
**Serum NLR**	0.906	0.728-1.128	0.37

* P < 0.05. CI, confidence interval; CRP, C-reactive protein; HR, hazard ratio; NLR, neutrophil-to-lymphocyte ratio.

## Discussion

This study revealed the potential predictive role of serum CRP and NLR levels in determining malignant kidney pathology after the renal mass biopsy procedure. We found that the parameters were significantly higher in malignant renal masses. These findings come as no surprise because of the previous associated data in the literature.

During malignancy, tumoral growth affects the homeostasis of human body and leads to an inflammatory response (6). CRP is a nonspecific acute-phase reactant associated with tissue damage, and serum concentration depends upon the synthesis rate. It is a simply measurable and useful indicator of systemic inflammation (7). Elevated serum CRP levels often reflect the pathological processes in the human body and are associated with several diseases including chronic inflammatory pathologies (7, 8). Recent evidences revealed that elevated serum CRP level was associated with several human cancers. Moreover, it had a prognostic role in most of the cancers including lung, pancreatic, and kidney cancers (7, 9–11). The relationship between CRP and cancer may occur with some biological mechanisms including tissue inflammation secondary to tumor growth and immune response to tumor antigens (12). Our results are consistent with the literature reporting the role of CRP in cancer-associated events. In our study, it established a significant increase in malignant masses. Moreover, it has a predictive role in determining the biopsy results. In the literature, the accepted cutoff value of CRP for investigation of several cancer-associated events was reported as 10 mg/L (6, 12). Therefore, we investigated the predictive role of serum CRP at a cutoff value of 10 mg/L. When the cutoff value was accepted as 10 mg/L, it predicted the malignant masses before the biopsy with a sensitivity and specificity of 76.6 and 81.8%, respectively. Moreover, it had a remarkable positive predictive value of 95%, whereas the negative predictive value was 39%. According to the above findings, we conclude that serum CRP measurement can be used as a promoter tool during indication and decision-making of the renal mass biopsy. In our opinion, a serum CRP level higher than 10 mg/L acts as an indicator to urologists and clinicians performing the renal mass biopsy. However, patients with lower serum CRP levels and a renal mass may be managed individually.

As such CRP, NLR is among the inexpensive and reproducible markers of systemic inflammation (13). The importance of NLR in several cancers has also been reported in the literature. It has been reported as a prognostic factor in lungs, gastric, colon, and kidney cancers (13, 14). Elevated NRL is an indicator of impaired cell-mediated immunity associated with systemic inflammation (15). Interaction between neutrophils and lymphocytes during inflammatory responses plays a critical role in carcinogenesis. The NLR reflects the balance between the activation of the inflammatory process and the antitumor immunity (14). The mechanism that underlies the association between elevated NLR and poor cancer prognosis has not been elucidated. However, aggregated systemic inflammatory response during the tumoral aggressiveness and cancer progression may lead to an increase in neutrophil counts and NLR (16). In the present study, similar to serum CRP levels, increased NLR was detected in malignant masses after the renal mass biopsy. Several cutoff values were reported for predictive roles of NLR in cancer patients in the literature. Here, we considered 2.1 as the cutoff value according to most of the associated reports (17–19). When the cutoff value was accepted as 2.1, it predicted the malignant masses before the biopsy with a sensitivity and specificity of 88.3 and 45.4%, respectively. The positive and negative predictive values were 89 and 41%, respectively.

Because of the positive significant correlation of serum CRP and NLR levels with malignant mass diameter, we performed univariate and multivariate cox regression analyses in terms of determining their independent predictive roles. Our findings revealed that serum CRP was an independent predictor in determining the malignant renal mass after the biopsy.

The major limitations of our study were the retrospective nature of the protocol and the small sample size. We did not use the specific cutoff levels for the parameters due to the small sample size. The effective cutoff value establishment could not be performed statistically. Instead of ineffective values, we used the reported values in the literature. Additionally, nonspecific elevation of serum CRP and NLR levels might have affected the findings. However, our study was the first to investigate the predictive factors for renal mass biopsy outcomes.

## Conclusion

In conclusion, pre-biopsy serum CRP and NLR levels were significantly different in patients with malignant pathology compared to those with benign pathology. They have a substantial diagnostic value in the diagnosis of malignant renal pathologies. Serum CRP, in particular, had a remarkable predictive role in determining the malignant renal pathologies after the renal mass biopsy. Therefore, pre-biopsy serum CRP and NLR levels may be used to predict the diagnostic outcomes of renal mass biopsy procedure. Further studies with larger cohorts may prove our findings in the future.

## References

[1] Sahni VA, Silverman SG. Biopsy of renal masses: When and why. Cancer Imag. 2009;9(1):44–55. 10.1102/1470-7330.2009.0005 PMC2739685 19602467

[2] Gray RE, Harris GT. Renal cell carcinoma: Diagnosis and management. Am Fam Physician. 2019;99(3):179–84. 30702258

[3] Campi R, Stewart GD, Staehler M, Dabestani S, Kuczyk MA, Shuch BM, et al. Novel liquid biomarkers and innovative imaging for kidney cancer diagnosis: What can be implemented in our practice today? A systematic review of the literature. Eur Urol Oncol. 2021;4(1):22–41. 10.1016/j.euo.2020.12.011 33408053

[4] Herts BR, Baker ME. The current role of percutaneous biopsy in the evaluation of renal masses. Semin Urol Oncol. 1995;13(4):254–61. 8595548

[5] Caoili EM, Davenport MS. Role of percutaneous needle biopsy for renal masses. Semin Intervent Radiol. 2014;31(11):20–6. 10.1055/s-0033-1363839 24596436 PMC3930651

[6] Hart PC, Rajab IM, Alebraheem M, Potempa LA. C-reactive protein and cancer-diagnostic and therapeutic insights. Front Immunol. 2020;11:595835. 10.3389/fimmu.2020.595835 33324413 PMC7727277

[7] Shrotriya S, Walsh D, Nowacki AS, Lorton C, Aktas A, Hullihen B, et al. Serum C-reactive protein is an important and powerful prognostic biomarker in most adult solid tumors. PLoS One. 2018;13(8):e0202555. 10.1371/journal.pone.0202555 30138391 PMC6107177

[8] Emery P, Gabay C, Kraan M, Gomez-Reino J. Evidence-based review of biologic markers as indicators of disease progression and remission in rheumatoid arthritis. Rheumatol Int. 2007;27(9):793–806. 10.1007/s00296-007-0357-y 17505829

[9] He X, Li JP, Liu XH, Zhang JP, Zeng QY, Chen H, et al. Prognostic value of C-reactive protein/albumin ratio in predicting overall survival of Chinese cervical cancer patients overall survival: Comparison among various inflammation based factors. J Cancer. 2018;9(10):1877–84. 10.7150/jca.23320 29805715 PMC5968777

[10] Shinohara S, Sugaya M, Onitsuka T, Machida K, Matsuo M, Tanaka F. Prognostic impact of postoperative C-reactive protein for non-small cell lung cancer following lobectomy. Anticancer Res. 2018;38(5):3193–8. 10.21873/anticanres.12584 29715162

[11] Ko YJ, Kwon YM, Kim KH, Choi HC, Chun SH, Yoon HJ, et al. High-sensitivity C-reactive protein levels and cancer mortality. Cancer Epidemiol Biomarkers Prev. 2012;21(11):2076–86. 10.1158/1055-9965.EPI-12-0611 23136255

[12] Heikkila K, Ebrahim S, Lawlor DA. A systematic review of the association between circulating concentrations of C reactive protein and cancer. J Epidemiol Community Health. 2007;61(9):824–33. 10.1136/jech.2006.051292 17699539 PMC2703800

[13] Kuo C, Hsueh WT, Wu YH, Yang MW, Cheng YJ, Pao TH, et al. The role of pretreatment serum neutrophil-to-lymphocyte ratio in hypopharyngeal cancer treated with definitive chemoradiotherapy: A pilot study. Sci Rep. 2019 7;9(1):1618. 10.1038/s41598-018-38282-z PMC6367463 30733592

[14] Faria SS, Fernandes PC, Jr., Silva MJ, Lima VC, Fontes W, Freitas-Junior R, et al. The neutrophil-to-lymphocyte ratio: A narrative review. Ecancermedicalscience. 2016;10:702. 10.3332/ecancer.2016.702 28105073 PMC5221645

[15] McMillan DC. Systemic inflammation, nutritional status and survival in patients with cancer. Curr Opin Clin Nutr Metab Care. 2009;12(3):223–6. 10.1097/MCO.0b013e32832a7902 19318937

[16] Templeton AJ, Ace O, McNamara MG, et al. Prognostic role of platelet to lymphocyte ratio in solid tumors: A systematic review and meta-analysis. Cancer Epidemiol Biomarkers Prev. 2014;23(7):1204–12. 10.1158/1055-9965.EPI-14-0146 24793958

[17] Chen Y, Chen K, Xiao X, Nie Y, Qu S, Gong C, Su F, Song E. Pretreatment neutrophil-to-lymphocyte ratio is correlated with response to neoadjuvant chemotherapy as an independent prognostic indicator in breast cancer patients: A retrospective study. BMC Cancer. 2016;16:320. 10.1186/s12885-016-2352-8 27198767 PMC4872336

[18] Azab B, Camacho-Rivera M, Taioli E. Average values and racial differences of neutrophil lymphocyte ratio among a nationally representative sample of United States subjects. PLoS One. 2014;9(11):e112361. 10.1371/journal.pone.0112361 25375150 PMC4223021

[19] Son SH, Park EY, Park HH, Kay CS, Jang HS. Pre-radiotherapy neutrophil-to-lymphocyte ratio as an independent prognostic factor in patients with locally advanced hepatocellular carcinoma treated with radiotherapy. Oncotarget. 2017;8(10):16964–71. 10.18632/oncotarget.15209 28199977 PMC5370014

